# Perception in autism does not adhere to Weber’s law

**DOI:** 10.7554/eLife.42223

**Published:** 2019-03-04

**Authors:** Bat-Sheva Hadad, Sivan Schwartz

**Affiliations:** 1 Department of Special EducationUniversity of HaifaHaifaIsreal; 2 Edmond J. Safra Brain Research CenterUniversity of HaifaHaifaIsrael; 3 Department of PsychologyUniversity of HaifaHaifaIsrael; University of OxfordUnited Kingdom; New York UniversityUnited States

**Keywords:** Autism, perception, Weber's law, Human

## Abstract

Perceptual atypicalities are a widely acknowledged but poorly understood feature of autism. We demonstrate here a striking violation of one of the most adaptive psychophysical computations – Weber’s law – in high-functioning individuals with autism. JNDs based on the best-fitting psychometric functions were measured for size visual judgments (Exp. 1), weight haptic discrimination (Exp. 2), and illusive perception of weight (brightness-weight illusion; Exp. 3). Results for the typically developed group confirmed Weber’s law, demonstrating a linear increase in JNDs with intensity, resulting in constant fractions across intensities. The results for the ASD, in contrast, showed no scaling of JNDs with intensity; instead, fractions decreased linearly with intensity. In striking contrast to its consistency in typical perception, Weber’s law does not hold for visual and haptic perception in autism. These robust modulations in psychophysical computations, demonstrated for different domains of perception, suggest a modality-independent, low-level mechanism driving altered perception in autism.

## Introduction

Autism Spectrum Disorder (ASD) refers to a group of neurodevelopmental disorders of yet unknown etiology. Sensory and perceptual atypicalities are a widely acknowledged but poorly understood feature of autism. Classic hypotheses on the possible origins of perceptual deficits in ASD, the weak central coherence theory and the enhanced perceptual functioning theory suggest that a local processing bias or processing style leads to the modulated perception [e.g., 1–2]. A Bayesian account has recently been posited to suggest a unifying computational framework by which internal priors are underweighted (Pellicano and Burr, 2012) or generate prediction errors that are estimated with high inflexible precision in ASD (Van de Cruys et al., 2014). The underlying assumption in many of these models is that sensory processing itself is intact (despite the emphasis on sensory abnormalities in ASD; see Van de Cruys et al., 2014; Brock, 2012), and changes in bottom-up factors are considered quantitative, mostly referring to changes in the amount of noise (see Brock, 2012). Other theories move beyond cognitive accounts, suggesting that an increased ratio of neural excitation and inhibition may give rise to autism symptomatology (e.g., Rubenstein and Merzenich, 2003; Rosenberg et al., 2015). Consistent with this hypothesis, it has been recently claimed that a reduction in the amount of inhibition through divisive normalization may lead to reduced influence of the nearby population of neurons on the activity of individual neurons (Rosenberg et al., 2015). According to this approach, there may be a link between divisive normalization and alterations in high-level functioning in autism, specifically involving a reduced influence of context on the interpretation of incoming sensory information. Here, we examined possible alternations in the adherence of perception in autism to Weber’s law by which perceptual resolutions are normally calibrated by the immediate context of the stimuli.

Sensory encoding is calibrated according to psychophysical principles defining physical-perceptual relations. During stimulus encoding, the perceptual system constructs and continually updates a generative model of the sensory inputs it receives. A robust manifestation of this transient plasticity is Weber’s law, given as Δ*I/I* = *C*, where Δ*I* is the increase in stimulus intensity to a level *I* that is required to produce a detectable change in intensity, and *C* is a constant. Sensitivity to changes in the intensities of the incoming input is thus relative, so that, for example, when a person is holding a five-pound weight, adding one pound will probably matter, but when that person is holding a fifty-pound weight, another added pound is much less likely to be noticed. This principle in perception is widely held to offer numerous functional advantages (e.g., Baird and Noma, 1978), including auto-calibrating the perceptual system to the environment by tuning its sensitivity to relative metrics. The auto-calibration, in turn, maximizes the use of the limited processing range of the system, disregarding minor and insignificant changes in the input while amplifying sensitivity to significant information. In typical perception, these computations have been shown to be strikingly similar across different sensory modalities, suggesting a common processing mechanism that characterizes human adults' perceptions in virtually all sensory dimensions (e.g., Baird and Noma, 1978; Ganel et al., 2008), emerging early in life (e.g., Hadad et al., 2012).

Despite their primary role, these computations are almost entirely overlooked in autism, even though there are some indications of altered calibration mechanism of stimulus encoding in this population. More particularly, the admittedly sparse evidence demonstrates reduced adaptation for auditory tones (Lawson et al., 2015), facial identity (Pellicano et al., 2007), numerosity stimuli (Turi et al., 2015), and touch (Tannan et al., 2008), indicating a reduced influence of an associated context on a nearby stimulus. Hypersensitivity to sensory stimuli and biographical reports about the apparent lack of habituation to stimuli with repeated exposure ([Bibr bib9][Bibr bib10]), the lack of contextual interference (e.g., Foxton et al., 2003), and findings demonstrating that perceptual representations remain close to the input (e.g., Hadad et al., 2017) all suggest difficulties using past experiences to derive expectations of incoming sensory signals; they also suggest reduced auto-calibration of the perceptual system to its environment (Pellicano and Burr, 2012).

We asked whether this reduced calibration in autism is evident at early stages of encoding during which sensitivity to changes in incoming stimulus is calibrated based on the immediate standard stimulation. Modulations in this calibration would suggest sensitivity to absolute rather than to relative metrics of the incoming input and might point to the very early stages of encoding as possible underlying mechanisms of altered perception. We employed three different types of magnitude judgement. Experiment one investigated possible modulations in the JND-intensity function for size reproduction in the visual domain, and Experiment two investigated weight discrimination in the haptic domain. These physical-perception relations were examined across the visual and haptic modalities to determine whether a modality-independent, low-level mechanism underlies the violation of basic psychophysics in autism. Experiment three extended the investigation to conditions where JNDs scale with the *perceived* rather than the *physical* intensities.

## Results

### Experiment 1: Size reproduction

**Figure 1.**
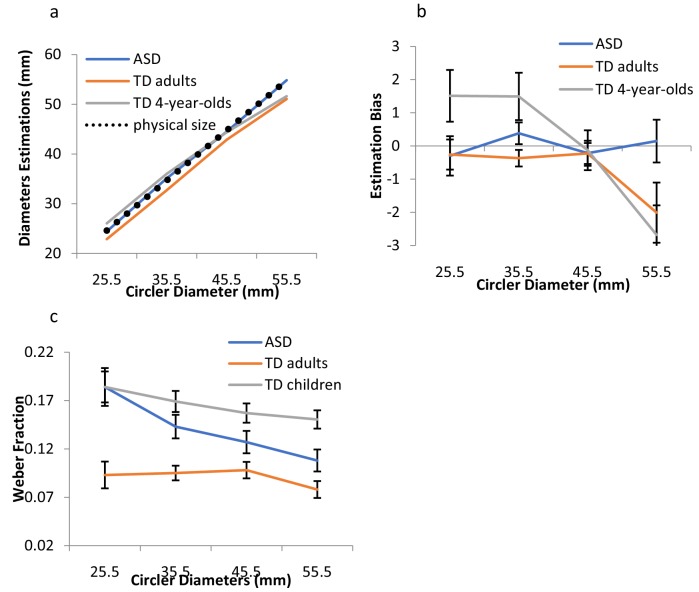
Results for Exp.1. (**a**) Mean estimated sizes for the different diameters for ASD individuals, TD adults, and TD children. The black dashed line indicates the real physical size. (**b**) The mean estimation bias (see text for details) is shown for the three groups. Estimations were biased towards the individual mean of estimations more for TD adults and the typically developing children than for the ASD individuals. (**c**) Weber’s fractions, computed as the within-subject SDs of estimations divided by the mean estimations for each size, are depicted as a function of the circler diameters, for the two groups. Adherence to Weber was indicated by the constancy of the coefficient of variation, normalized to the perceived magnitude. Weber fractions remained constant across the different sizes, as predicted by Weber, for the TD children and adults, but not for those with ASD. Error bars indicate 95% within-subjects CI. 10.7554/eLife.42223.003Figure 1—source data 1.Source data for Exp. 1 - Size estimation task.

Participants were asked to perceptually reproduce the size of a 2-D circle varying in diameter using the method of adjustment. Four different circles (diameters of 25.5, 35.5, 45.5, and 55.5 mm) were presented, one at a time. JNDs for a given size were computed in terms of the within-participant standard deviations (SDs) of the perceptual estimates for each size (Ganel et al., 2008). JNDs reflect the ‘area of uncertainty’ for which the observer is insensitive to the difference between the size of the comparison and the target object (Ganel et al., 2008). Weber’s law (i.e., the minimum detectable increment in magnitude increases proportionally with stimulus magnitude) predicts proportional increments in JNDs with object size (Hadad et al., 2017; Baird and Noma, 1978; Ganel et al., 2008). To correct for perceptual biases in size perception (see differences in estimations and actual size in Figure 1a), estimations of each size were computed correcting for biases in the overall mean of estimations for each individual, in each of the groups. Specifically, for each participant, the correction was computed as the following: (estimated size – physical size) - (perceived grand mean- physical grand mean). Results for the raw estimation and the response bias in estimations are presented in Figure 1a and b. Below we report the results for the corrected estimations (the analysis on the raw estimations, demonstrating a similar pattern of results, is reported in Appendix 1 and in Appendix 1—figure 1). Data from one participant were excluded from analysis because his bias-corrected estimates were below 2.5 standard deviations of the group mean. The pattern of the results remains the same with and without his data.

### ASD and age- and IQ-matched TD adults

A mixed-design ANOVA with size as a within-subject factor and group (TD adults vs. ASD) as a between-subject factor was carried out on observers’ estimations. As can be seen in Figure 1a, mean estimations generally scaled with the physical size of the circles, *F*(3,132) = 954.01, p<0. 0001, η^2^_p_ =.96. However, the analysis on the mean differences between physical and estimated sizes showed that individuals with autism were more accurate than typical observers (TDs), *F*(1,44) = 5.65, p<0.022, η^2^_p_ =.12. Overall, the latter underestimated the circle diameters. Interestingly, a mixed-design ANOVA carried out on observers’ corrected estimations revealed a significant effect for group, *F*(1,44) = 7.44, p<0.009, η^2^_p_ =.15, indicating that while the TD’s estimations demonstrated ‘regression to the mean,’ mostly indicated by underestimation of the larger sizes, those of the ASD observers exhibited a much smaller bias (Figure 1b).

For Weber’s fractions, the main measures of interest, the results revealed a striking difference between the TD and the ASD observers (see Figure 1c). Weber’s fractions were computed for each individual, in each of the diameters, as the SD of estimation divided by the mean estimation. A mixed-design ANOVA with diameter size as a within-subject factor and group as a between-subject factor revealed a significant interaction between diameter size and group, *F*(3,132) = 2.94, p<0.036, η^2^_p_ =.11. (This interaction between group and diameter size on Weber’s fractions remains significant when the outlier data are included, *F(*3,135)=3.05, p<0.03).) For the TD observers, Weber’s fractions remained fairly constant across the different diameters, *F*(1,19) = 2.84, p>0.11, indicating a clear adherence to Weber’ s law. For the ASD observers, in contrast, there was a linear decrease in the fractions with disc diameters, *F*(1,25) = 13.15, p<0.001, η^2^_p_ =.35, demonstrating a clear violation of Weber’s law.

### TD children and adults

When we compared the estimations of TD adults with those of typically developing children, we discovered Weber’s law was already apparent in the young group. A mixed-design ANOVA with diameter size as a within-subject factor and group (TD adults vs. TD children) as a between-subject factor was carried out on observers’ estimations. As can be seen in Figure 1a, mean estimations generally scaled with the physical diameters, *F*(3,105) = 481.42, p<0.0001, η^2^_p_ =.94. A mixed-design ANOVA on the corrected estimations, (estimated size – physical size) - (perceived grand mean- physical grand mean), revealed a significant effect of group, *F*(1,35) = 4.81, p<0.035, η^2^_p_ =.12, indicating larger biases in the children’s group. Both adults and children demonstrated ‘regression to the mean’; however, the children’s bias was larger than the adults’ bias, in both overestimating the smaller sizes and underestimating the larger sizes (Figure 1b; see Duffy et al., 2006 for similar results).

For Weber’s fractions, the results revealed a very similar pattern between TD adults and typically developing children (Figure 1c). A mixed-design ANOVA was carried out on Weber’s fraction (SD of estimations/mean estimations), with size as a within-subject factor and group as a between-subject factor. The analysis revealed a significant effect of group, *F*(1,35) = 50.97, p<0.0001, η^2^_p_ =.59, demonstrating, as expected, larger fractions and overall lower sensitivity in children. The interaction between group and size did not reach significance, *F*(3,105) = 1.56, p>0.20. JNDs scaled significantly with the circle diameters, *F*(3,105) = 11.36, p<0.0001, η^2^_p_ =.23, in adherence to Weber, regardless of age, *F* < 1. We found linear components for both young children and adults, η^2^_p_ =.23, and η^2^_p_ =.24, respectively, demonstrating the adherence of visual perception to Weber’s law as early as four years of age (see Figure 1a in Appendix 1 – Figure 1).

Critically, the flat function depicting the object size–JND relations in ASD (indicated by the decreased fractions with size) cannot be attributed to poor resolution, difficulties in task understanding, or ceiling effects. First, estimations provided by the observers with autism were more accurate than those provided by the TD observers (Figure 1a), indicating enhanced resolution in size estimations. Second, Weber fractions (Figure 1c), indicative of discrimination resolution, were not associated whatsoever with adherence to Weber’s law: fractions were greater in TD children than in either ASD or TD adults, but scaled with the disc diameters in complete adherence to Weber. The differences in the shape of the function in the ASD group thus suggest modifications in the way visual information is encoded and calibrated during the very basic levels of processing.

### Experiment 2: From vision to touch – Is Weber’s law also violated for haptics in ASD?

To determine the extent to which the violation of basic psychophysics in autism reflects a general, low-level alert mechanism, Experiment two extended the examination of Weber to the somatosensory domain, testing the perception of weight (Namdar et al., 2016) In a procedure using the method of constant stimuli, four standards of weights were presented (300, 350, 400, and 450 g), with 12 different weights for each standard, varying in steps of 6 g. Participants were presented with two plastic water containers and were asked to indicate which was heavier. The frequency with which each comparison stimulus was judged heavier than the standard was plotted against the values of the comparison stimuli for each standard. The resulting psychometric data were fitted by a sigmoid for each standard stimulus for each participant. The JNDs were then calculated by identifying the stimulus values corresponding to 25% and 75% of comparisons chosen as heavier and then halving the difference. (Interestingly, responses on the lower part of each psychometric function (discriminating between each standard weight and its lighter comparisons) were noisier that those on the upper part of these functions. Noisier responses for the lower part of the function could be the result of a conflict between the perceptual representations and the response, in a manner similar to that found in other commonly employed perceptual paradigms (see, for example, (Hadad and Kimchi, 2018; Zemel et al., 2002). In our case, a conflict may have arisen between the response required (‘which is heavier?') and the perceptual representations of the comparisons (i.e., whether comparisons are indeed heavier). Because the standard was more extensively presented and was therefore likely to serve as a reference (‘anchoring’; e.g., Harris, 1948), participants were most probably evaluating the comparisons relative to this standard value. In cases where the comparisons were indeed heavier (at the upper part of the function), the response was compatible with the perceptual representation. Otherwise, a conflict emerged, leading to noisier responses.)

**Figure 2.**
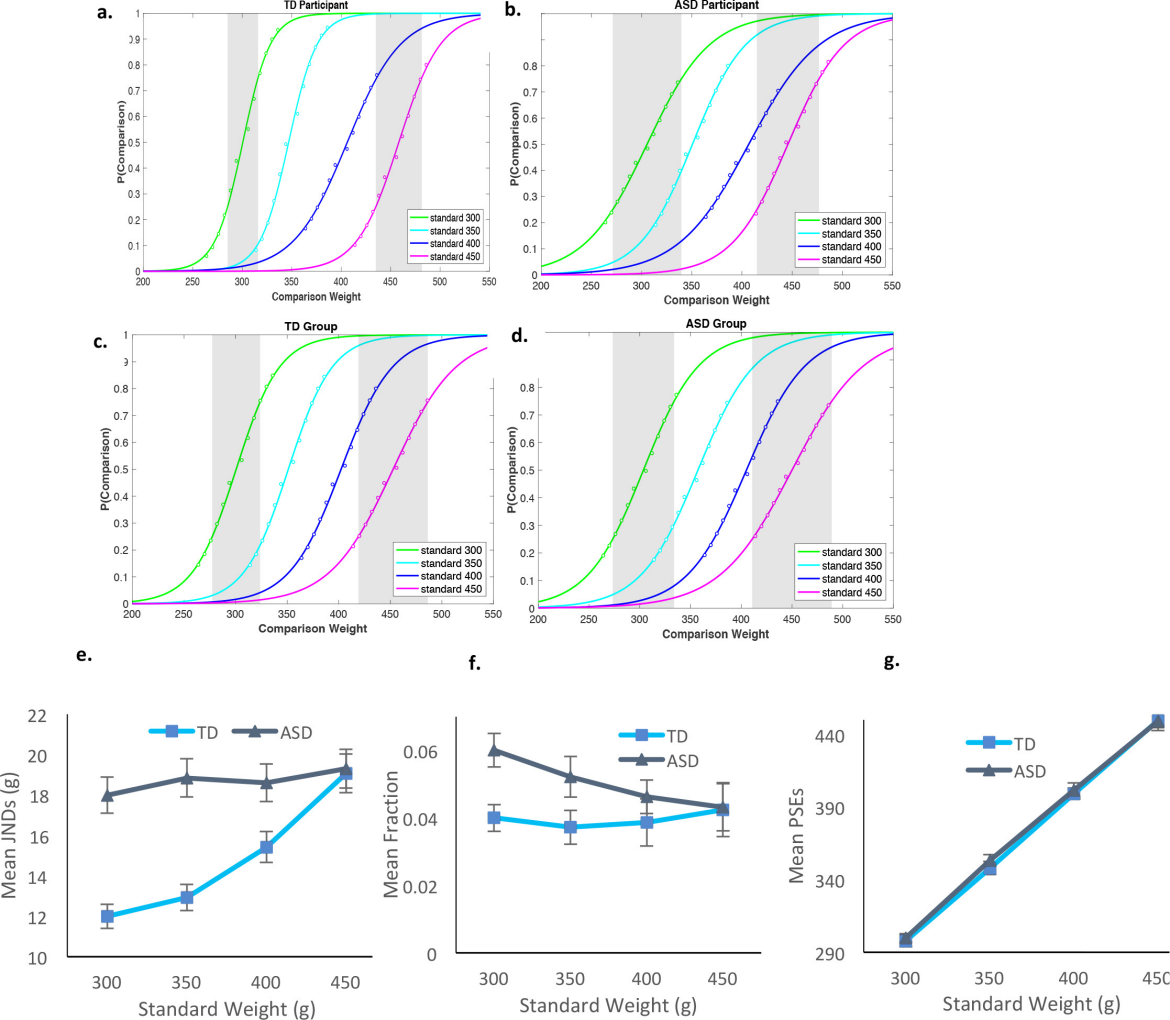
Results for Exp. 2. Psychometric functions, plotting the proportion of trials in which participants reported the comparison as heavier, as a function of weight of the comparisons. The different functions refer to the four standards. (**a**) Data for a representative TD participant. (**b**) Data for a representative individual diagnosed with ASD. (**c**) All data for the typical group pooled (n = 11). (**d**) All data for the ASD group pooled (n = 12). The shaded gray areas in each graph signify JNDs for standards of 300 and 450 g. (**e**) JNDs increased linearly with weight only for the TD group. (**f**) Weber’s fractions were computed as the ratio of the JNDs to the PSEs (JNDs/PSEs), computed for each weight for each participant. These fractions did not remain constant as a function of weight for ASD, violating Weber. (**g**) PSEs demonstrated accurate perception in both groups. As in the visual estimation task, TD adults showed a subtle tendency to underestimate the standard weights. Error bars in e-f indicate standard errors.

Figure 2a–d present the psychometric functions of a typical and an autistic participant and of the collapsed functions across participants in each group. Figure 2e–2g present the computed JNDs, Weber fractions, and PSEs for the different standards for the two groups (the complete set of individual psychometric functions appears in Figure 2—figure supplement 1 and Figure 2—figure supplement 2). Preliminary analysis of the function deviations (squared distances between actual data points and the sigmoid predictions) revealed no significant differences in the reliability of functions between the groups, across all standards, t(21)=1.05), p>0.3, for each of the standards [for 300 g: *t*(21)=1.2, p>0.23; for 350 g: *t*(21)=0.05, p>0.9; for 400 g: *t*(21)=1.8, p>0.07, and for 450 g: *t*(21)=0.32, p>0.7]. The analysis on the PSEs suggested that the different weights were similarly perceived by the TD and the ASD individuals, with no significant interaction between weight and group, *F* < 1 (Figure 2g).

**Figure 2—figure supplement 1.**
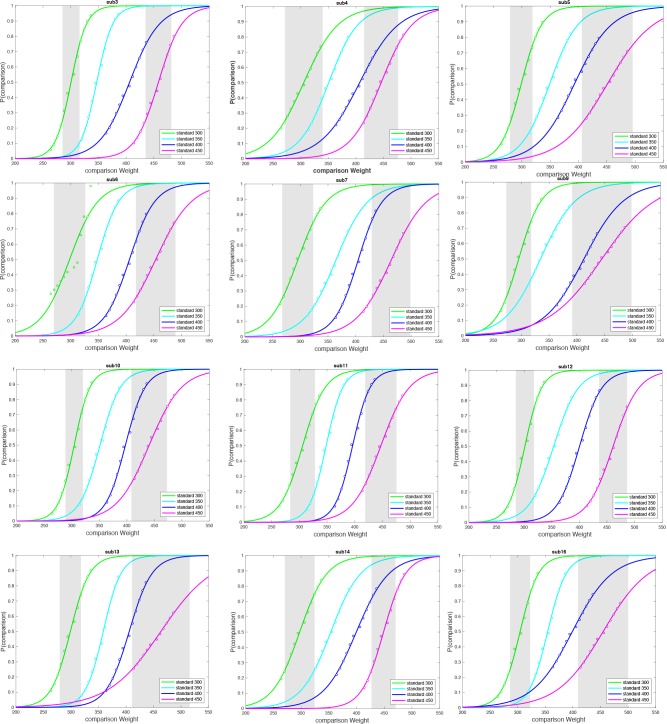
Individual Psychometric functions for the TD group.

**Figure 2—figure supplement 2.**
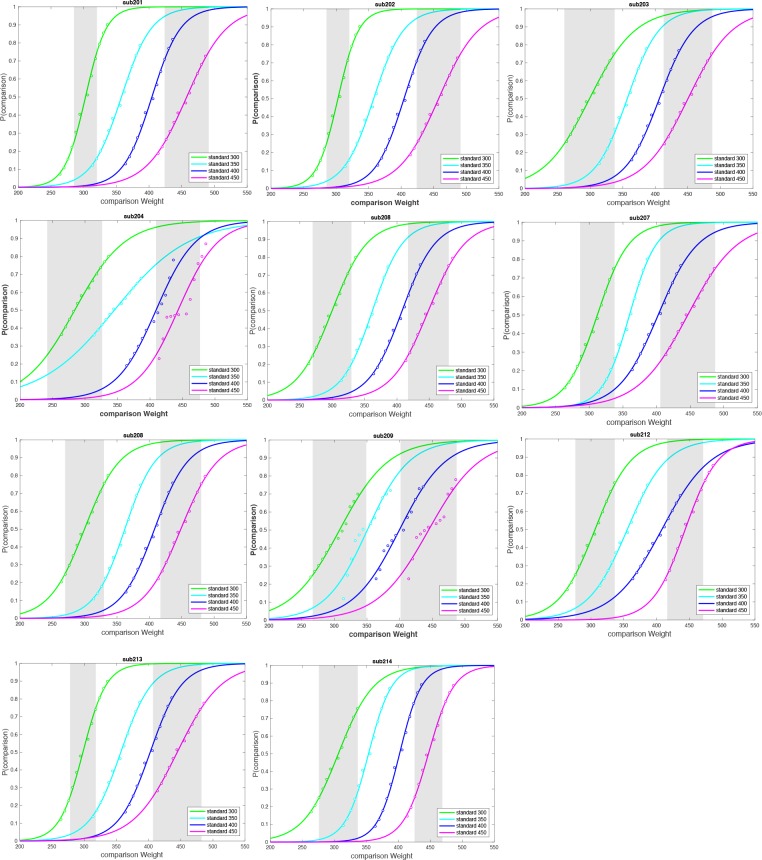
Individual Psychometric functions for the ASD group.

The results for the Weber’s fractions replicated the pattern we found in the visual domain, demonstrating adherence to Weber only in the TD group (Figure 2). In the main analysis, we calculated Weber fractions when normalized to the perceived weight, computed as JNDs/PSE, and also in terms of the physical weight, computed as JNDs/standard weight. A mixed-design ANOVA was carried out on these Weber fractions, with group as a between-subject factor and the standard weight as a within-subject factor. The analysis revealed a significant interaction between group and the standard weight for both the JNDs/PSE and the JNDs/standard weight fractions, *F*(3,63) = 3.13, p<0.03, η^2^_p_ =.13, *F*(3,63) = 3.17, p<0.03, η^2^_p_ =.13.

Further analysis of this interaction confirmed Weber’s law, with fractions remaining constant across the different weights for the TD group, *F* < 1. For the autistic group, Weber’s fractions decreased linearly with increased weight, *F*(3,33) = 2.87, p<0.05, η^2^_p_ =.20, replicating the pattern observed for the visual domain. Specific comparisons testing the effects of weight on JNDs in each group showed that while for the ASD group, JNDs did not scale with weights, *F* < 1, for the TD group they did, *F*(3,30) = 3.50, p<0.027, η^2^_p_ =.26, with a significant linear component best characterizing their JND-intensity function, *F*(1,10) = 7.21, p<0.023, η^2^_p_ =.42.

### Experiment 3: Weber’s law for illusive intensities

**Figure 3.**
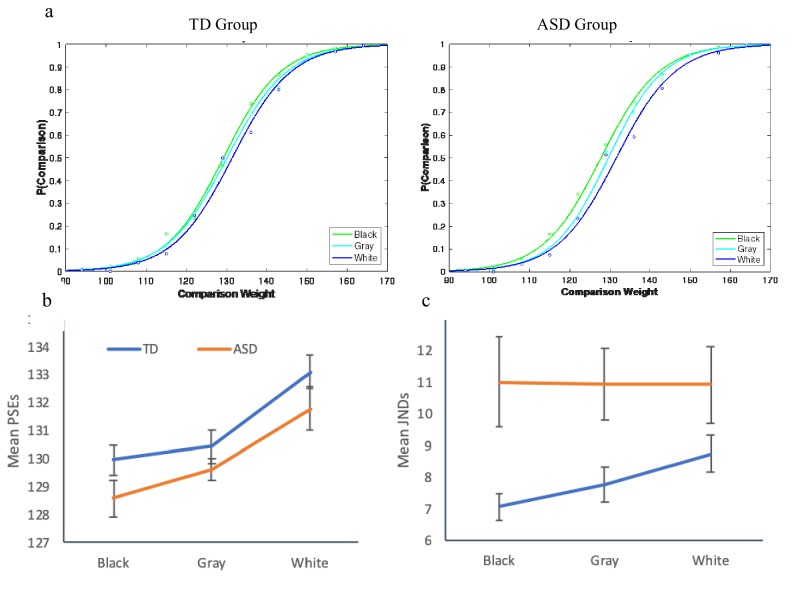
Results for Exp. 3. (**a**) Psychometric functions, plotting the proportion of trials for which participants reported the comparison as heavier, as a function of the brightness of the standards. Both groups were affected by the illusion, overestimating the weight of the white standard and underestimating the weight of the black standard. (**b**) Both groups exhibited larger PSEs for the white standard and smaller PSEs for the black standard, compared to the gray control standard. (**c**) Only the TD group demonstrated scaling of the JNDs to the perceived weight, in adherence to Weber’s law. Individuals with ASD exhibited constant JNDs across the perceived weights.

Having demonstrated the lack of scaling of JNDs with *physical* intensities in ASD, we performed a third experiment to query a possible violation of Weber for *perceived* intensities. Specifically, the adherence to Weber was tested for illusive intensities by examining susceptibility to the brightness-weight illusion (Walker et al., 2010). In this illusion, darker objects, when *observed*, are judged heavier than brighter but otherwise identical objects (presumably because of the statistics associating surface lightness and weight; e.g., a wet log is often darker and heavier). However, when darker and lighter objects are *lifted and observed,* the opposite pattern often appears, presumably because of a correction unconsciously employed, based on implicit expectations (Walker et al., 2010). We tested TD adults (n = 21) and adults diagnosed with high-functioning autism (n = 18), all with IQs within the normal range, in a weight discrimination task using a method of constant stimuli. The standards weights were 129 gr containers colored in gray, white, or black. For each of these standards, we created a set of comparisons composed of gray containers varying in steps of 7 g. Cumulative Gaussian distribution functions were computed for each individual, plotting the proportion of trials in which the comparison was reported as heavier as a function of weight of the comparisons. For both groups, the points of subjective equality (PSEs) of the white container were shifted to the right, indicating the illusive perception of the white container as heavier. An inspection of the individual functions in the three conditions indicated that the majority of the participants in both the ASD and the TD groups exhibited susceptibility to the illusion. The critical relevant question, however, was whether JNDs of the weight discriminations scaled with the illusive perceived weights. We thus computed individual JNDs in each of the conditions (gray, black, or white standards) for those individuals in each group who showed a shift in PSEs for the black and the white standards from the gray one, resulting in a group of 12 ASD and 16 TD individuals. A mixed-design ANOVA with brightness as a within-subject factor and group as a between-subject factor was carried out on the PSEs and JNDs, derived from the individual psychometric functions. A significant effect of brightness on PSEs was found, *F*(2,52) = 16.06, p<0.0001, η^2^_p_ =.38, that did not interact with group, *F* < 1. This effect of brightness showed a significant linear trend, *F*(1,26) = 17.32, p<0.0001, η^2^_p_ =.40 (Figure 3b), indicating a shift in PSEs between the functions, with smaller PSEs for black than gray, and larger PSEs for white than gray (significant linear trends appeared for both TD *F*(1,15) = 16.08, p<0.0001, η^2^_p_ =.50, and ASD *F*(1,11) = 4.66, p<0.05, η^2^_p_ =.33). Interestingly, a mixed-design ANOVA on the JNDs revealed a marginally significant interaction between group and standard weight, *F*(2,52) = 3.19, p<0.07, η^2^_p_ =.09. JNDs scaled with the *perceived* weight for the TD individuals, *F*(2,30) = 3.69, p<0.05, η^2^_p_ =.35, demonstrating a significant linear trend, *F*(1,15) = 7.73, p<0.014, η^2^_p_ =.34. Again, in a clear contrast to the TD participants, no effect of brightness on JNDs was found for the ASD group, *F* < 1. In other words, for the TD individuals, JNDs scaled with the *perceived* intensities as predicted by Weber’s law, but for the ASD individuals, JNDs did not scale with the perceived weights. The results of this experiment strengthen the findings of Experiments 1 and 2 and extend them to suggest a violation of Weber and the atypical sensitivity of ASD individuals to the absolute changes in the incoming stimulation for *perceived* intensities, not just *physical* ones.

## Discussion

The results show that perception in autism, in both the visual and haptic domains, does not adhere to Weber’s law. Individuals with autism encode changes in the incoming input in absolute terms, demonstrating JNDs that, in clear contrast to those of TD individuals, do not scale with object magnitudes. Importantly, this violation of Weber is strikingly similar across different modalities and perceptual domains, suggesting common low-level altered mechanisms and possibly offering a parsimonious modality-independent explanation of the altered perception in autism.

The scaling of perceptual sensitivity (i.e., JNDs) with the magnitude of the stimulation, as defined by Weber, is widely held to offer numerous functional advantages (e.g. Ganel et al., 2008), including auto-calibrating the perceptual system to the environment by tuning its sensitivity to relative metrics. This auto-calibration normally makes maximal use of the limited processing range of the system, disregarding minor and insignificant changes in the input while amplifying sensitivity to significant information. The violation of Weber in autism demonstrates disruptions in such processes, producing perceptual representations that remain closer to the input. Our finding is consistent with the recently reported reduced adaptation effects (Lawson et al., 2015; Turi et al., 2015) but suggests that not only does perception in autism not calibrate itself to past experience and related context but it also shows no immediate lower-stream calibration based on the stimulation itself (Rakitin et al., 1998). The recording of incoming changes in absolute metrics may lead to the sensory overload often reported in these individuals (e.g., O'Donnell et al., 2012).

Our results provide important insights into the Bayesian account of atypical perception in autism. According to this approach, perception results from a variable weighting of the sensory input in relation to the prior, such that higher sensory uncertainty (noisier or more difficult stimuli) often lead to less weighting of the likelihood (sensed magnitude) and to stronger top-down modulations (e.g., Petzschner et al., 2015). The effects of perceptual priors may be stronger for larger magnitudes presumably because of increased uncertainty in the input. The lack of scaling of uncertainty in the measurement with the object magnitude we found here for individuals with autism may therefore change the variable weighting of the sensory input in relation to the prior, suggesting that the mechanism underlying the underuse of priors in this group may be related to the way the incoming sensory input is registered: the fairly constant noise in the encoded incoming input (i.e., likelihood) leading to the constant JNDs across stimulus magnitude could change the way the likelihood and its precision influence the relative weight and the utilization of the prior, leading to the modulations seen in the perceptual processing of those with autism.

In terms of the *predictive coding* theory of autism, which is equivalent to the Bayesian approach but replaces sensory evidence with predication errors such that incoming information is put in context from the very start (Van de Cruys et al., 2014), modulations in Weber’s law may provide evidence for the claim that individuals with ASD show inflexibly high precision in their prediction errors. The mechanism by which the perceptual system constructs and continually updates a generative model of the sensory inputs it receives (i.e., Weber’s function) meets an inflexible sensory system in ASD that minimalizes deviations from the actual incoming sensory input. Although determining the exact mechanism must await direct manipulation of likelihood and priors, our results suggest that modulations in the likelihood must be taken into consideration when taking a Bayesian approach to study perceptual atypicalities in autism.

The neural mechanism underlying the perceptual sensitivity to relative changes in the environment in accordance with Weber is unknown. It may be related to the canonical neural normalization that computes a ratio between the response of an individual neuron and the summed activity of a pool of neurons (Carandini and Heeger, 2011). Normalization produces an analogous context dependence, where the neural representation of the value is explicitly dependent on the value of other available alternatives. It has been suggested as a mechanism that maximizes sensitivity by facilitating discrimination among representations of different stimuli and reducing redundancy. Normalization has also been proposed to be at the root of the modulatory effect of attention and other higher-order processes, such as multi-sensory integration on neural responses of various functions in the cortex. Through neural network simulations, a compelling explanation has been recently provided for the perceptual symptoms of autism in terms of a failure of divisive normalization (Rosenberg et al., 2015). The findings suggest that a reduction in the amount of inhibition that occurs through divisive normalization can account for perceptual consequences of autism, consistent with the hypothesis of an increased ratio of neural excitation to inhibition (E/I) in these individuals. These disturbances in the mechanisms that normally serve to contextualize neural responses are consistent with the current results demonstrating reduced calibration of changes in incoming input, and with the claim of high precision of sensory evidence in this population.

Finally, because of the fundamental nature of these computations, the modulations we documented in individuals with autism may be closely associated with their sensory symptoms. This qualitatively modulated psychophysics demonstrates disproportional responsivity to changing incoming stimulation, such that the JND-intensity function they exhibit and the deviation of this function from linear may result in larger JNDs than neurotypicals in cases of small but not larger magnitudes. In fact, if Weber’s law works in neurotypicals for larger magnitudes than those used here, smaller JNDs may be expected for ASD individuals, possibly demonstrating ASD hypersensitivity to the same stimulation for which these same individuals exhibit hyposensitivity and elevated thresholds. Such a pattern may account for seemingly inconsistent findings of differences in perceptual processing between ASD and TD individuals: individual with ASD may show elevated thresholds for low intensities but comparable and even enhanced performance than for neurotypicals at higher intensities. The results may also account for the different subtypes of sensory abnormalities shown along intensities within the same individual [e.g., hyposensitivity (higher JNDs) to some intensities but hypersensitivity to others along the same dimension of stimulation]. Combined with individual thresholds and measures of sensory symptomatology in ASD individuals, this psychophysics may be used to identify signatures of atypical psychophysics that may, in turn, predict the symptoms of ASD and define different subtypes of sensory abnormalities (e.g., hypo- or hypersensory responders).

The results demonstrate qualitative modulations in basic psychophysical processes serving the fundamental function of transient plasticity in ASD, where the output of perceptual processes depends on absolute changes rather than on their calibration based on the immediate standard stimulation. Modulations occurring within basic computations determining the resolution power of discriminating changes in the incoming input may account for the atypical perception demonstrated in the higher-level processing and for the sensory symptoms of autism.

## Materials and methods

### Exp. 1

#### Participants

Sixty-three persons in three groups participated in the experiment: 20 TD adults (mean age = 26.5; range = 24–30 years; 13 females), 26 adults diagnosed with ASD (mean age = 21.5; range = 18–28 years; eight females), and 17 TD children aged 4–5 years (mean age = 4.8; range = 3.9–5.1 years; 11 females; see Table 1) (We set the sample size in advance to match the sample size we typically use in our studies measuring perceptual processes in autism employing within-subject designs. Past experience suggests this sample size is sufficient to show clear differences between groups [e.g., Hadad and Ziv, 2015; Hadad et al., 2017; Hadad and Kimchi, 2018]). Participants had normal or corrected to normal vision.

**Table 1.** Participants’ details, IQ scores, and AQ scores for Experiment 1

	N (Female, Male)	Age (range)	Approx. IQ (range; SD)	AQ (range; SD)
ASD	26 (8,18)	21.5 (18–28)	107.73 (82-125; 15.37)	23.21 (16-39; 8.16)
TD Adults	20 (12,8)	26.5 (24–30)	117.35 (95-120; 8.25)	16.50 (5-22; 4.80)
TD Children	17 (11,6)	4.8 (3.9–5.1)	-	-

ASD diagnosis was confirmed by previous diagnoses based on the ADOS-G (Lord et al., 2000). Participants also completed the short Hebrew version of the Wechsler Intelligence Scale (WAIS-III/WISC-IV) to assess IQ. The short version (Kaufman et al., 1996) includes both verbal and performance intelligence tests shown to yield reliable scores in ASD (Minshew et al., 2005). The approximated IQ scores were obtained by summing the scores of all four tests, multiplying the sum by 1.6, and adding 36 (Lord et al., 2000). The IQ scores of the ASD group did not differ from those of the typical group, t(43)=1.56, p>0.15.

Each participant (or participant’s guardian, when required) provided written informed consent. TD participants were recruited through advertisements at the university, and ASD participants were recruited through hostels, assisted living centers, and Internet forums. All procedures were approved by the Ethical Committee of the Faculty of Education, University of Haifa. Adults who were students at the University of Haifa received course credit, children received a gift card, and individuals with ASD were paid 50NIS per hour to compensate them for their time.

#### Stimuli and procedure

The experimental protocol was approved by the local Research Ethics Committee. The procedures were explained, and informed consent was obtained from the adult participants and from the parents of the children. At a viewing distance of approximately 50 cm, participants were asked to adjust a comparison 2-D circle, presented on one side of the computer display, to match a standard 2-D circle appearing on the other side of the screen by using the mouse wheel. Four disc sizes (from a sitting distance of 60 cm, the diameters subtended visual angles of 2.43, 3.39, 4.34, and 5.30°) were presented, one at a time. The diameter of the comparison circle varied across trials with two possible sizes randomly determined for each trial. One was 10 pixels smaller than the smallest standard, and the other 10 pixels larger than the larger standard. Presentation order was pseudorandomized, and each size was repeated 16 times. Participants were asked to respond as accurately as possible. Six practice trials were given before the actual experiment to ensure participants understood the task.

### Exp. 2

#### Participants

Twenty-three new participants in two groups participated in the experiment: 12 TD adults (mean age = 23.6; range = 20–26 years; four females) and 11 adults diagnosed with ASD (mean age = 28.3; range = 23–30 years; three females; see Table 2). (We set the sample size to match the sample size we typically use in our studies measuring full individual psychometric functions in autism [Hadad et al., 2017] Participants had normal or corrected to normal vision.

**Table 2.** Participants’ details, IQ scores, and AQ scores for Experiment 2

	N (Female, Male)	Age (range)	Approx. IQ (range; SD)	AQ (range; SD)
ASD	11 (3,8)	28.3 (23–30)	105.3 (80–120; 12.56)	25.32 (13-39; 9.21)
TD adults	12 (4,8)	23.6 (20–26)	115.35 (95-120; 7.24)	14.50 (6-22; 5.20)

#### Stimuli and procedure

The stimuli were plastic water containers of the same size. They were filled with different volumes of water to create the standards and comparisons weight stimuli. The method of constant stimuli was used, with four values serving as standards for the determination of the JNDs: 300, 350, 400, and 450 g. The containers were opaque so that vision of the level of water was blocked. The participant sat in front of a table, at a distance that allowed both elbows to be placed on the edge of the table. In each trial, the participant was presented with two containers in rapid sequence, the standard stimulus and a comparison stimulus, and was asked to indicate verbally which of the two containers was heavier. The containers were placed approximately 15 cm apart on the table, with their positions relative to each other determined randomly for each trial. The comparison stimuli were 12 different weights for each standard, varying in steps of 6 g. For example, the comparison stimuli for the standard of 400 g varied between 364 and 436 g. Participants were asked to lift each container only once to a height of approximately 10 cm, using both hands with their elbows not touching the table. Each pair of containers (one of the four standards coupled with one of the comparisons) was repeated 12 times, resulting in 144 trials per standard and a total of 576 trials. Participants were tested in two or three separate sessions; each session lasted approximately two hours. The standards and comparisons were mixed within each session and were randomly selected. Participants could take a break after every 36 trials and were required to take a five-minute break after every 72 trials. Breaks were administered to rest the participant's arm muscles and to optimize engagement in the task.

### Exp. 3

#### Participants

Thirty-nine participants in two groups participated in the experiment: 21 TD adults (mean age = 24.6; range = 19–27 years; seven females) and 18 adults diagnosed with ASD (mean age = 24.2 range=21–28 years; five females; see Table 3). A small group of participants in each group also participated in Experiment 2. Participants had normal or corrected to normal vision. The data of 16 TD individuals and 12 ASD individuals in this group who were susceptible to the illusion were analyzed to test for adherence to Weber’s law.

**Table 3.** Participants’ details, IQ scores, and AQ scores for Experiment 3 (only for participants who were susceptible to the illusion)

	N (Female, Male)	Age (range)	Approx. IQ (range; SD)	AQ (range; SD)
ASD	12 (4,8)	25.3 (21–28)	110.3 (85–120; 12.26)	27.12 (14-39; 9.21)
TD adults	16 (6,10)	25.6 (19–27)	112.15 (95-120; 8.24)	15.50 (7-22; 5.20)

#### Stimuli and procedure

The stimuli were plastic water containers of the same size, filled with different volumes of water to create the standards and comparisons weight stimuli. The method of constant stimuli was used, where three containers with the equal weight of 129 g, colored in three brightness levels—black, gray, and white—served as standards for the determination of the PSEs and JNDs. The comparison containers for each of these standards were all gray. The containers were opaque so that participants’ vision of the level of water was blocked. The participant sat in front of a table, at a distance that allowed both elbows to be placed on the edge of the table. In each trial, the participant was presented with two containers in rapid sequence, the standard stimulus and a comparison stimulus, and was asked to indicate verbally which of the two containers was heavier. The containers were placed approximately 15 cm apart on the table, with their positions relative to each other determined randomly for each trial. The comparison stimuli were 11 different weights for each standard, varying in steps of 7 g. Participants were asked to lift each container only once to a height of approximately 10 cm, using both hands, with their elbows not touching the table. Each pair of containers (one of the three standards coupled with one of the comparisons) was repeated 12 times, resulting in 132 trials per standard and a total of 396 trials. Participants were tested in two or three separate sessions; each session lasted approximately an hour. The standards and comparisons were mixed within each session and were randomly selected. Participants could take a break after every 36 trials and were required to take a five-minute break after every 66 trials. Breaks were administered to rest the participant's arm muscles and to optimize engagement in the task.

### Data analysis

In Experiment 1, the within-subject variability of estimations produced by the participants served as the main dependent measure. Within-participant standard deviations (SDs) of the perceptual estimates for each disc size were computed for each individual for each of the sizes. These SDs are found to indicate the JNDs for a given size, reflecting an ‘area of uncertainty’ within which the observer is insensitive to the difference between the size of the comparison and the target object (Hadad et al., 2012). Weber’s law (i.e., minimum detectable increment in stimulus magnitude increases proportionally with stimulus magnitude) predicts linear increments in JNDs with object size (Baird and Noma, 1978; Ganel et al., 2008).

In Experiments 2 and 3, the frequency with which each comparison stimulus was judged heavier than the standard was plotted against the values of the comparison stimuli for each standard. Sigmoid functions were fitted to the mean subject data of each individual. JND values were determined by specifying the stimuli for 25% and 75% ‘heavier’ responses and halving the difference.

## Figures and Tables

**Appendix 1—figure 1.**
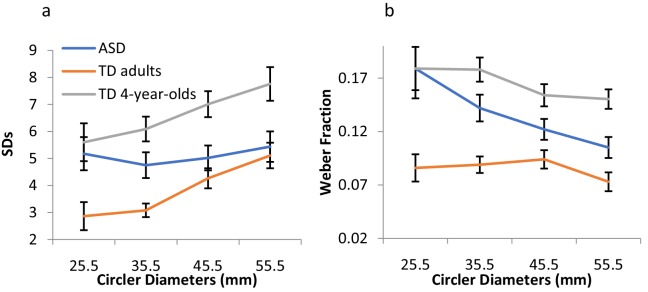
Exp. 1 results - raw estimations. (**a**) The standard deviations (JNDs) of the raw estimations as a function of the diameters for ASD individuals, TD adults, and TD children. (**b**) Weber’s fractions, computed as the within-subject SDs of estimations divided by the physical sizes, remained constant across the different sizes, as predicted by Weber, for the TD groups only. Error bars indicate 95% within-subjects CI.

## Data Availability

Data can be found in https://osf.io/ckmhq/. The following dataset was generated: HadadB-S2018Weber in AutismOpen Science Frameworkckmhq

## Additional files

Transparent reporting form

## Appendix 1

### Exp. 1. Analysis for raw estimations

10.7554/eLife.42223.012 ASD and age- and IQ-matched controls Weber’s fraction (Δ*I/I*), computed as the within-subject SDs of estimations divided by the actual diameters, confirmed the violation of Weber in the autistic group. A mixed-design ANOVA on Weber fractions with diameter size as a within-subject factor and group as a between-subject-factor revealed a significant interaction between group and diameter size, *F*(3,132)=3.25, p<0.024, η^2^_p_ =.07. The fractions for the ASD group decreased linearly with diameter size, *F*(1,25) = 12.23, p<0.002, η^2^_p_ =.33, but remained fairly constant across size for the TD group, *F*(1,19) = 1.81, p>0.19, in complete adherence to Weber (Figure 1b). TD children and adults A mixed-design ANOVA on Weber fractions with size as a within-subject factor and group as a between-subject-factor revealed an expected decrease in Weber fractions with age, *F*(1,35) = 58.01, p<0.0001, η^2^_p_ =.62. However, the interaction between group and size did not reach significance, *F*(3,105) = 2.34, p>0.08, indicating rather constant fractions with size for both groups.
