# Supplementary material for: Perception in autism does not adhere to Weber’s law
Source: eLife. 2019 Mar 4;8:e42223. doi: 10.7554/eLife.42223 (PMC6420264; doi:10.7554/eLife.42223)
Supplement: Peer review history

# Decision letter

**DOI:** 10.7554/eLife.42223.017

**Affiliations:** New York UniversityUnited States; University of FlorenceFlorenceItaly

In the interests of transparency, eLife includes the editorial decision letter and accompanying author responses. A lightly edited version of the letter sent to the authors after peer review is shown, indicating the most substantive concerns; minor comments are not usually included.

Thank you for submitting your article "Perception in autism does not adhere to Weber's law" for consideration by *eLife*. Your article has been reviewed by Timothy Behrens as the Senior Editor, a Reviewing Editor, and three reviewers. The following individual involved in review of your submission has agreed to reveal his identity: David Charles Burr (Reviewer #2).

The reviewers have discussed the reviews with one another and the Reviewing Editor has drafted this decision letter to help you prepare a revised submission.

In this novel study, the authors obtain psychophysical data in three experiments to assess the presence or absence of empirical support for Weber's law in individuals with ASD and in typically developing individuals for basic perceptual judgments – size and weight reproduction or dis-crimination. The authors report that Weber's law fails in autism, suggesting atypical normalization mechanisms.

All reviewers agree that this is an interesting, novel study. However, there are a number of serious questions and concerns regarding methodological and theoretical issues. Addressing the following points will provide information regarding the validity of the claims and strengthen the manuscript. If substantiated, this is a finding with potentially profound implications. There are very few "laws" in perception; thus, results showing that an atypical population exhibits no Weber-type dependence would be a significant finding.

The reporting of the data analysis and results needs to be greatly improved,

Theoretical issues:

1) This study is really about lack of normalization (as has been shown for adaptation), yet his term appears only late in Discussion section; it would be useful to include it in the motivation. Cite the relevant paper by Rosenberg et al., (2015).

2) Describe the meaning of Weber's law in the Introduction.

2.1) In the Introduction, the assumption that sensory processing in ASD is intact is mentioned. That was the case 5-10 years ago, but now there is a consensus against this assertion. See the review by Robertson and Baron-Cohen, (2017).

3) The discussion is hard to follow and sometimes self-contradictory. For example, the Weber's law is described as a low-level sensory property (which is correct), but then the authors also link it to unconscious inference and top-down modulations (Discussion section).

4) If substantiated, the reported finding has potentially profound implications, which is mentioned only at the end of the manuscript. This point should be elaborated in a more prominent place. The finding that Weber's law fails in autism could help explain a number of seemingly conflicting behavioral results in the ASD literature: The relative difference in perceptual performance between TD and ASD would depend on the baseline stimulus value chosen – with individuals with ASD being worse for low intensity stimuli (e.g., low weight), have typical performance for medium intensity stimuli and perform better for high intensity stimuli. This last point is related to a methodological concern (1). In any case, the authors should either show (see below) or at least mention the prediction of better performance at the high end of stimulus values.

Materials and methods section and Results section:

1) The authors report no evidence for the Weber's law in ASD (for the range of stimulus values used). The narrow range of stimulus values used -a two-fold range of visual sizes and a 1.5-fold range of weights- is enough for a proof of principle. However, to make a strong conclusion for no Weber's law in ASD one would like to have a wider range of stimulus values. Note that for both visual size (Figure 1C) and weight (Figure 2F) the data for ASD and TD converge for the highest intensity stimuli. What happens for even higher intensity stimuli would very diagnostic for the authors' main conclusion. If there is indeed no Weber's law in ASD, then the prediction is that individuals with ASD should do better-than-typical for even higher intensity stimuli. Where the observed trend continues in the reported data (i.e., no Weber type scaling), there would be better than normal performance in ASD with a few higher level stimulus values. The impact of this paper would be much higher with a wider range of stimulus levels, as the authors would show both impairment and enhancement in the same task, and be able to explain these results with a simple common explanation. We recommend the authors conduct an experiment including a wider range of stimulus levels. However, acknowledging that this is a study with an atypical population and that it may not be easy to conduct another experiment, at least we'd like the authors to acknowledge that they used a narrow range and to explain why they chose such a restricted range.

2) Weber fraction is technically JND/PSE: that is, thresholds are normalized to perceived rather than to physical size. JND/Intensity is the coefficient of variation. It has become common practice to use Weber as a proxy for CoV, but this is an issue here as the authors wish thresholds to vary with perceived, illusory size (which is Weber). The authors should make very clear what they are doing here to avoid confusion. It would be acceptable to state they choose to call CoV Weber fraction in keeping with modern practice.

3) AQ scores for the ASD group are unusually low: mean of 23, is much lower than the ex-pected mean for ASD (~35; Ruzich et al. (2015) Measuring autistic traits in the general population: a systematic review of the Autism-Spectrum Quotient (AQ) in a nonclinical population sample of 6,900 typical adult males and females).

4) Experiment 1, Methods:

4.1) How was the starting point of the comparison circle set? Was it always smaller or larger than the standard or randomly determined? Could the starting point of the diameter be related to the consistent underestimation in the TD group.

4.2) Is there a reason participants were instructed to respond as quickly and accurately as possible? I understand the accuracy, but why quickly? Might heightened time pressure lead to increased trial-to-trial variability?

4.3) Make more explicit that way that the Weber Fraction estimates are computed in Figure 1C, using the language from other Experiment 1 variables.

5) Experiment 1, Results:

5.1) The statistical reporting in Experiment 1 is rather vague. Multiple F statistics are re-ported, but there is no explanation of the exact test used (one-way, mixed-design, etc.) and no explanation of the factors. Reporting of test and factors, like in subsection “Experiment 3: Weber’s law for illusive intensities”, is needed for Experiment 1. It is hard to interpret the statistical results without this information; for example, is the claim that there was greater accuracy in ASD, relative to TD observers (subsection “ASD and age- and IQ-matched controls”), supported by a main effect of group in a mixed-design ANOVA on the difference between mean diameter produced and objective diameter? Is this for all TD ob-servers or only the adults? What does the F<1 in subsection “ASD and age- and IQ-matched controls”refer to?

5.2) The statistics seem to be incorrectly interpreted sometimes. It is reported that a significant interaction indicates, "as expected, more accurate estimations for the older observers" (subsection “TD children and adults”). But according to Figure 1A, it appears that the 4-year old participants are more accurate than the TD adults, for all points tested except the 55.5 diameter (i.e., the 4 year-olds lie closer to the physical line for all points but the last one). Why are the TD adults consistently underestimating (i.e., all points lie below the physical size line)? Why are they less accurate than the 4 year-olds TD participants? These serious concerns raise questions about the appropriateness of the adult TD group as a comparison for the ASD group. Add error bars and data markers in the figure.

6) Experiment 2:

6.1) More information on the analysis is needed. The JND metric is described in subsection “Experiment 2: From vision to touch – Is Weber’s law also violated for haptics in ASD?”, and also in subsection “Data analysis”, but then a footnote seems to invalidate that description. Were the JNDs based only on the upper part of each psychometric function (Footnote 1) or using the formula provided in the text in subsection “Experiment 2: From vision to touch – Is Weber’s law also violated for haptics in ASD?”? If only the upper part was used, the justification for doing so does not seem adequate. This needs to be addressed before a complete assessment of Experiment 2 can be completed.

6.2) Experiment 2 is 2AFC yielding a cumulative Gaussian, from which they took 25 and 75% points. Why not use standard deviation (σ), so is can relate directly to the measurement of Experiment 1?

6.3) Regarding Figure 2 and Figure 2—figure supplement 1 and Figure 2—figure supplement 2, why do the "representative subject" functions in Figure 2 appear to be the least well fit by the functions? The data points fall very neatly on or close to the fit line in the supplementary figures, for almost all subjects, but not at all in Figure 2.

7) Experiment 3:

7.1) The data presented in Figure 3A also raise questions. The fits in both figures look pretty bad given that these are pooled across participants. Also, and more critical, how is it the case that all of the data points lie only at proportions that are evenly spaced (i.e., 1, 0.9, 0.8, 0.7), with no intermediate points? Even if the number of trials dictate that an individual subject's data could only be at these values (for example if there are only 10 trials per condition), when averaged across 18 and 21 participants, it is almost impossible to get data points that are always separated by 0.1.

7.2) Again, the statistical results are hard to evaluate because it is not clear what was done. Subsection “Experiment 3: Weber’s law for illusive intensities” says, "A mixed-design ANOVA with brightness as a within-subject factor and group as a between-subject factor was carried out on the PSEs and JNDs." Then a significant main effect and no interaction are presented for the PSE data. But when the JND results are presented, it is not clear what each F statistic refers to. Was there an interaction? Significant main effects? It reads as if two separate ANOVAs were completed one for the TD group and one for the ASD group, but that would contradict the information presented elsewhere in subsection “Experiment 3: Weber’s law for illusive intensities”.

[Editors' note: further revisions were requested prior to acceptance, as described below.]

Thank you for resubmitting your work entitled "Perception in autism does not adhere to Weber's law" for further consideration at *eLife*. Your revised article has been favorably evaluated by three reviewers. The manuscript has been improved. The reviewers feel that you have satisfactorily addressed many issues, but concerns remain regarding the data analysis and some missing methodological details, as outlined below. These are important, as they are the bases for the discussion and conclusions.

Please make sure to provide all methodological details and explain clearly the logic of the analysis. We will make the final decision on your next revised manuscript, without further iterations.

- In Experiment 1 of the revised manuscript you dropped a participant from the TD adult group, who was included in the original version, because of "overall extreme underestimation". You should provide a quantitative justification for doing so. Would the conclusions be the same if this participant were included?

- Are the data presented in Figure 1A the actual diameter estimations or the "bias-corrected" estimations referenced in the text? You should provide the justification of this bias-corrected estimation procedure, which was not mentioned in the previous version.

- Are the Weber fractions depicted in Figure 1C computed using the bias-corrected estimates? Are the results the same without this correction? The reported values seem to have increased (roughly doubled?) in this version – Is that due to the bias-correction procedure?

- It reads as if the "bias-correcting" procedure is a response to a previous question/concern about the TD group consistently underestimating. For the data to be interpretable, it is necessary to know whether and why this bias correction procedure is necessary only for the TD group (subsection “ASD and age- and IQ-matched TD adults”). How can you assure readers that the results do not hinge on this correction?

- Looking at Figure 1B in the revised version, it seems that the bias really only exists at the 55.5 data point, but based on Figure 1A, it seems as if the adult TD group is consistently underestimating.

- Figure for Experiment 2 also changed (though the caption was not marked as changed). Explain how the change came about.

# Reply: Author response

**DOI:** 10.7554/eLife.42223.018

In this novel study, the authors obtain psychophysical data in three experiments to assess the presence or absence of empirical support for Weber's law in individuals with ASD and in typically developing individuals for basic perceptual judgments – size and weight reproduction or dis-crimination. The authors report that Weber's law fails in autism, suggesting atypical normalization mechanisms.

All reviewers agree that this is an interesting, novel study. However, there are a number of serious questions and concerns regarding methodological and theoretical issues. Addressing the following points will provide information regarding the validity of the claims and strengthen the manuscript. If substantiated, this is a finding with potentially profound implications. There are very few "laws" in perception; thus, results showing that an atypical population exhibits no Weber-type dependence would be a significant finding.

The reporting of the data analysis and results needs to be greatly improved,

Theoretical issues:

1) This study is really about lack of normalization (as has been shown for adaptation), yet his term appears only late in discussion; it would be useful to include it in the motivation. Cite the relevant paper by Rosenberg et al., (2015).

We have edited the Introduction to include the hypothesis of lack of normalization in ASD; in addition to the reduced adaptation findings as part of our motivation for the study.

2) Describe the meaning of Weber's law in the Introduction.

We have edited the Introduction to include the definition and meaning of Weber’s law at the outset, as suggested.

2) In the Introduction, the assumption that sensory processing in ASD is intact is mentioned. That was the case 5-10 years ago, but now there is a consensus against this assertion. See the review by Robertson and Baron-Cohen, (2017).

We agree. We have edited this paragraph to clarify that this claim refers specifically to Weber’s law and to processes determining the physics-sensation relations which have not been tested but are generally assumed to be typical in ASD by most Bayesian models.

3) The Discussion section is hard to follow and sometimes self-contradictory. For example, the Weber's law is described as a low-level sensory property (which is correct), but then the authors also link it to unconscious inference and top-down modulations.

We have edited the Discussion section. We hope this editing clarifies our interpretation of the results. We agree that adherence to Weber’s law is a low-level sensory characteristic; in fact, this was the main motivation for our research. However, we now also discuss our results in terms of the predictive coding theory of autism (Van de Cruys et al., 2014), suggesting that modulations in Weber’s law may be taken as direct evidence that individuals with ASD show inflexibly high precision in their prediction errors.

4) If substantiated, the reported finding has potentially profound implications, which is mentioned only at the end of the manuscript. This point should be elaborated in a more prominent place. The finding that Weber's law fails in autism could help explain a number of seemingly conflicting behavioral results in the ASD literature: The relative difference in perceptual performance between TD and ASD would depend on the baseline stimulus value chosen – with individuals with ASD being worse for low intensity stimuli (e.g., low weight), have typical performance for medium intensity stimuli and perform better for high intensity stimuli. This last point is related to a methodological concern (1). In any case, the authors should either show (see below) or at least mention the prediction of better performance at the high end of stimulus values.

We now emphasize the implications of the violation of Weber in autism for understanding the seemingly inconsistent findings of modulations in perceptual processing in individuals with ASD, and also for understanding their sensory symptoms. In addition, we explicitly discuss the possibility of comparable or even higher sensitivity (lower JNDs) in ASD for higher intensities than those used here.

Materials and methods section and Results section:

1) The authors report no evidence for the Weber's law in ASD (for the range of stimulus values used). The narrow range of stimulus values used -a two-fold range of visual sizes and a 1.5-fold range of weights- is enough for a proof of principle. However, to make a strong conclusion for no Weber's law in ASD one would like to have a wider range of stimulus values. Note that for both visual size (Figure 1C) and weight (Figure 2F) the data for ASD and TD converge for the highest intensity stimuli. What happens for even higher intensity stimuli would very diagnostic for the authors' main conclusion. If there is indeed no Weber's law in ASD, then the prediction is that individuals with ASD should do better-than-typical for even higher intensity stimuli. Where the observed trend continue in the reported data (i.e., no Weber type scaling), there would be better than normal performance in ASD with a few higher level stimulus values. The impact of this paper would be much higher with a wider range of stimulus levels, as the authors would show both impairment and enhancement in the same task, and be able to explain these results with a simple common explanation. We recommend the authors conduct an experiment including a wider range of stimulus levels. However, acknowledging that this is a study with an atypical population and that it may not be easy to conduct another experiment, at least we'd like the authors to acknowledge that they used a narrow range and to explain why they chose such a restricted range.

We definitely agree that extending these levels to the point where JNDs are smaller in ASD than in TD would be very informative. However, because Weber’s law is known to hold over the middle range of stimulus dimensions but fails near the extreme ends (e.g., Baird, 1997; Baird and Noma, 1978; Falmagne, 1986; Ward and Davidson, 1993), it was critical to stay within a range for which a clear scaling of JNDs with intensities has been demonstrated. The range of stimulus levels used in each of the experiments was chosen based on earlier data demonstrating proportional scaling of JNDs with intensities, indicating clear adherence to Weber in neurotypicals. We could not employ wider ranges, as testing already required long sessions of perceptual discrimination tasks (each experiment took two or even three separate long sessions).

In the Discussion section, we now consider the possibility that larger ranges of stimulation may have resulted in lower JNDs in ASD than in the TD group and that such a result could explain the hypersensitivity often reported in ASD even for the same stimulation to which hyposensitivity is shown.

2) Weber fraction is technically JND/PSE: that is, thresholds are normalized to perceived rather than to physical size. JND/Intensity is the coefficient of variation. It has become common practice to use Weber as a proxy for CoV, but this is an issue here as the authors wish thresholds to vary with perceived, illusory size (which is Weber). The authors should make very clear what they are doing here to avoid confusion. It would be acceptable to state they choose to call CoV Weber fraction in keeping with modern practice.

We now clarify that adherence to Weber is indicated by the constancy of the coefficient of variation, which was normalized to the perceived magnitude to control for differences in possible biases in the perception of magnitude.

3) AQ scores for the ASD group are unusually low: mean of 23, is much lower than the expected mean for ASD (~35; Ruzich et al., (2015) Measuring autistic traits in the general population: a systematic review of the Autism-Spectrum Quotient (AQ) in a nonclinical population sample of 6,900 typical adult males and females).

AQ is a self-administered questionnaire and thus could be less reliable and less variant within the ASD group. The main purpose of administering this measurement was to screen out those in the TD group who scored high on this measurement. ADOS scores were high for all participants diagnosed with ASD, and the original diagnosis was confirmed, even for those with unexpectedly lower AQ scores.

4) Experiment 1, Methods:

4.1) How was the starting point of the comparison circle set? Was it always smaller or larger than the standard or randomly determined? Could the starting point of the diameter be related to the consistent underestimation in the TD group.

The diameter of the comparison circle varied across trials, with two possible sizes randomly determined for each trial. One was 10 pixels smaller than the smallest standard and the other 10 pixels larger than the larger standard. We now explain this in the Method of Experiment 1.

4.2) Is there a reason participants were instructed to respond as quickly and accurately as possible? I understand the accuracy, but why quickly? Might heightened time pressure lead to increased trial-to-trial variability?

Participants were instructed to reproduce the size of the comparison circle as accurately as possible. The stimuli stayed on screen until responses were received. We apologize for the mistake and have corrected the manuscript.

4.3) Make more explicit that way that the Weber Fraction estimates are computed in Figure 1C, using the language from other Experiment 1 variables.

Corrected as suggested (subsection “TD Children and adults”).

5) Experiment 1, Results:

5.1) The statistical reporting in Experiment 1 is rather vague. Multiple F statistics are re-ported, but there is no explanation of the exact test used (one-way, mixed-design, etc.) and no explanation of the factors. Reporting of test and factors, like in subsection “Experiment 3: Weber’s law for illusive intensities”, is needed for Experiment 1. It is hard to interpret the statistical results without this information; for example, is the claim that there was greater accuracy in ASD, relative to TD observers (subsection “ASD and age- and IQ-matched controls”), supported by a main effect of group in a mixed-design ANOVA on the difference between mean diameter produced and objective diameter? Is this for all TD ob-servers or only the adults? What does the F<1 in subsection “ASD and age- and IQ-matched controls” refer to?

We have edited the statistical report for Experiment 1.

5.2) The statistics seem to be incorrectly interpreted sometimes. It is reported that a significant interaction indicates, "as expected, more accurate estimations for the older observers" (subsection “TD children and adults”). But according to Figure 1A, it appears that the 4-year old participants are more accurate than the TD adults, for all points tested except the 55.5 diameter (i.e., the 4 year olds lie closer to the physical line for all points but the last one). Why are the TD adults consistently underestimating (i.e., all points lie below the physical size line)? Why are they less accurate than the 4 year-olds TD participants? These serious concerns raise questions about the appropriateness of the adult TD group as a comparison for the ASD group. Add error bars and data markers in the figure.

The TD adults demonstrated a general bias of underestimating diameter sizes. To correct for this bias, we computed the individual estimations considering the overall mean of estimations for each individual. Specifically, the individual estimations for each size were computed as the following: (estimated size – physical size) – (perceived grand mean- physical grand mean). TD adults and children demonstrated a “regression to the mean” bias; and as expected, it was larger for the larger magnitudes (Petzschner, Glasauer and Stephan, 2015). Interestingly, the ASD data did not show this bias (Figure 1B).

6) Experiment 2:

6.1) More information on the analysis is needed. The JND metric is described in subsection “Experiment 2: From vision to touch – Is Weber’s law also violated for haptics in ASD?”, and also in subsection “Data analysis”, but then a footnote seems to invalidate that description. Were the JNDs based only on the upper part of each psychometric function (footnote 1) or using the formula provided in the text in subsection “Experiment 2: From vision to touch – Is Weber’s law also violated for haptics in ASD?”? If only the upper part was used, the justification for doing so does not seem adequate. This needs to be addressed before a complete assessment of Experiment 2 can be completed.

As in sequential discrimination tasks, enhanced perceptual resolutions appear when perceptual “anchoring” is established (stable internal reference gradually formed by repeated exposures (e.g., Ahissar, 2007). This may explain the differences we found in deviations in the lower versus the upper parts of the functions. However, based on the reviewer’s comment, we now report the findings from the whole function. We keep our original comment (Footnote #1) on this possible response competition.

6.2) Experiment 2 is 2AFC yielding a cumulative Gaussian, from which they took 25 and 75% points. Why not use standard deviation (σ), so is can relate directly to the measurement of experiment 1?

Experiments 2 and 3 both used constant stimuli in 2AFC tasks; thus, we computed thresholds in the more common manner. The data of the σ of these functions is presented below.

**Table:** 

group	standard	slope	deviations
TD	300	0.049257	0.034234
	350	0.046532	0.031874
	400	0.0389	0.027495
	450	0.032131	0.023567
ASD	300	0.037416	0.025416
	350	0.037156	0.026734
	400	0.034547	0.024798
	450	0.033375	0.02184

6.3) Regarding Figure 2 and Figure 2—figure supplement 1 and Figure 2—figure supplement 2, why do the "representative subject" functions in Figure 2 appear to be the least well fit by the functions? The data points fall very neatly on or close to the fit line in the supplementary figures, for almost all subjects, but not at all in Figure 2.

7) Experiment 3:

7.1) The data presented in Figure 3A also raise questions. The fits in both figures look pretty bad given that these are pooled across participants. Also, and more critical, how is it the case that all of the data points lie only at proportions that are evenly spaced (i.e., 1, 0.9, 0.8, 0.7), with no intermediate points? Even if the number of trials dictate that an individual subject's data could only be at these values (for example if there are only 10 trials per condition), when averaged across 18 and 21 participants, it is almost impossible to get data points that are always separated by 0.1.

We apologize for the mistakes in the figures. We have corrected them; all fitting procedures are now accurate.

7.2) Again, the statistical results are hard to evaluate because it is not clear what was done. Subsection “Experiment 3: Weber’s law for illusive intensities” says, "A mixed-design ANOVA with brightness as a within-subject factor and group as a between-subject factor was carried out on the PSEs and JNDs." Then a significant main effect and no interaction are presented for the PSE data. But when the JND results are presented, it is not clear what each F statistic refers to. Was there an interaction? Significant main effects? It reads as if two separate ANOVAs were completed one for the TD group and one for the ASD group, but that would contradict the information presented elsewhere in subsection “Experiment 3: Weber’s law for illusive intensities”.

We hope the paper now clearly presents the main findings of the interactive effects of group and brightness on JNDs but not on PSEs.

[Editors' note: further revisions were requested prior to acceptance, as described below.]

Thank you for resubmitting your work entitled "Perception in autism does not adhere to Weber's law" for further consideration at eLife. Your revised article has been favorably evaluated by three reviewers. The manuscript has been improved. The reviewers feel that you have satisfactorily addressed many issues, but concerns remain regarding the data analysis and some missing methodological details, as outlined below. These are important, as they are the bases for the discussion and conclusions.

Please make sure to provide all methodological details and explain clearly the logic of the analysis. We will make the final decision on your next revised manuscript, without further iterations.

- In Experiment 1 of the revised manuscript you dropped a participant from the TD adult group, who was included in the original version, because of "overall extreme underestimation". You should provide a quantitative justification for doing so. Would the conclusions be the same if this participant were included?

We added a quantitative justification (Subsection “ASD and age- and IQ-matched TD adults”). The pattern of the results remains the same with and without the outlier data (Footnote #1).

- Are the data presented in Figure 1A the actual diameter estimations or the "bias-corrected" estimations referenced in the text? You should provide the justification of this bias-corrected estimation procedure, which was not mentioned in the previous version.

Thank you for pointing this. We now make it clear that Figure 1A presents the actual diameters estimations and Figure 1B presents the bias-corrected estimations (Subsection “ASD and age- and IQ-matched TD adults”). We now also more clearly explain the bias-corrected estimation procedure and the reason for implementing this correction before reporting the ANOVA results (Subsection “ASD and age- and IQ-matched TD adults”). The pattern of the main findings is the same with or without the bias correction. We report the original analysis (without the bias correction) in the supplementary information (Appendix 1 and Appendix 1—figure 1).

- Are the Weber fractions depicted in Figure 1C computed using the bias-corrected estimates? Are the results the same without this correction? The reported values seem to have increased (roughly doubled?) in this version – Is that due to the bias-correction procedure?

We understand now that the way we responded to the previous comments was confusing. To clarify, the report and the figures presented in the manuscript are now based on the bias-corrected estimations, but the data without the correction (the original report and the figures) fare also presented in a supplementary file (Appendix 1 and Appendix 1—Figure 1).

The fractions originally presented were lower because SDs were divided by the radii sizes in pixels. We apologize for the confusion. Now, in both versions, the fractions are computed in terms of the diameters in millimeters. The pattern of results does not change between the two versions, both indicating constant fractions across the different sizes for TD but not for ASD. We have uploaded the data file (Figure 1—source data 1).

- It reads as if the "bias-correcting" procedure is a response to a previous question/concern about the TD group consistently underestimating. For the data to be interpretable, it is necessary to know whether and why this bias correction procedure is necessary only for the TD group (subsection “ASD and age- and IQ-matched TD adults”). How can you assure readers that the results do not hinge on this correction?

We now make it clear that the correction was done in all groups and that it does not change the main findings (Subsection “ASD and age- and IQ-matched TD adults”). Results in the main text are reported in terms of the corrected estimations. The analysis based on the raw estimations provided an identical pattern of results and is reported in the supplementary file.

- Looking at Figure 1B in the revised version, it seems that the bias really only exists at the 55.5 data point, but based on Figure 1A, it seems as if the adult TD group is consistently underestimating.

Figure 1A presents the actual estimations and the black dashed line indicates the real physical size. In comparison to the actual diameters, TD individuals indeed exhibited an overall underestimation of the sizes (Figure 1A); however, when corrected for the individual bias, TD adults mainly showed underestimation of the 55.5 mm size (Figure 1B).

- Figure for Experiment 2 also changed (though the caption was not marked as changed). Explain how the change came about.

We added a sentence to the figure caption that clarifies that Figure 2 also presents the PSEs for the different weights for the two groups (Figure 2G). We also explain the rational of doing so in subsection “Experiment 2: From vision to touch – Is Weber’s law also violated for haptics in ASD?”.
